# Experience with Renal Autotransplantation: Typical and Atypical Indications

**DOI:** 10.1155/2018/3404587

**Published:** 2018-03-26

**Authors:** Ali Bourgi, Rana Aoun, Elias Ayoub, Maroun Moukarzel

**Affiliations:** Department of Urology, Hotel-Dieu de France University Hospital, Boulevard Alfred Naccache, Achrafieh, P.O. Box 166830, Beirut, Lebanon

## Abstract

**Introduction and Objectives:**

Renal autotransplantation is a kidney-saving surgical procedure used in selected patients. The purpose of this report is to review nine typical and atypical indications for kidney autotransplantation and evaluate its effectiveness in maintaining kidney function and avoiding cancer recurrence.

**Materials and Methods:**

From 1999 till 2014, nine renal autotransplantations were performed in our center. A retrospective case review was done. Four of nine patients had a solitary functioning kidney. Typical indications for autotransplantation included extended ureteric disease in 5 patients, intrasinusal tumor on a solitary kidney in 1 patient, and renal artery aneurysm in 1 patient. Atypical indications consisted in bilateral urothelial tumors in 1 patient and interrupted live kidney transplantation in 1 patient. Mean cold ischemia time was 209 minutes. Demographic factors, indications, renal function before and after surgery, and in the long term, cancer recurrence and disease-free survival were evaluated.

**Results:**

Renal function was maintained in 8 patients during the early follow-up. No serious complications occurred in the postoperative period. Median duration of follow-up was 50 months. In 4 patients with a normal contralateral kidney, mean preoperative and at discharge creatinine clearance were 105.45 ml/min and 121.02 ml/min, respectively. Although values showed an improvement in the kidney function, the difference was not significant (*p* value 0.3). In the other 4 patients with a solitary kidney, mean discharge creatinine clearance was 99.24 ml/min surprisingly higher than the preoperative value 96.92 ml/min. At the last follow-up, kidney function was preserved for the two groups (normal contralateral kidney/solitary kidney) with relatively stable creatinine clearance values: 108.45 ml/min and 85.9 ml/min, respectively. No patients required secondary dialysis.

**Conclusion:**

Renal autotransplantation is a rare, safe, and effective surgical procedure for the treatment of complex urologic conditions. In some instances, it may be of great utility for kidney salvage in some carefully selected patients.

## 1. Introduction

Renal autotransplantation (RA) is a rare, safe, and effective surgical procedure for the treatment of complex urologic conditions. It was first reported by J. D. Hardy in 1963 when he repaired a high ureteric injury following aortic surgery by reimplanting the repaired organ into the ipsilateral iliac fossa.

Hardy's accomplishment was made possible, thanks to allotransplantation techniques developed by Dr Murray and colleagues in Boston a decade earlier. Managing renal disease via a “benchwork” approach soon became a novel idea pursued worldwide by surgeons [1].

After this landmark surgery, RA has been steadily improved to a safe and effective procedure and has been used in the treatment of different complex urologic diseases like extensive ureteric injuries, complex nephrolithiasis, loin-pain syndrome, renovascular diseases (stenotic lesions of distal renal arteries, intrarenal aneurysms, and arteriovenous malformations), tumors of the kidney and ureter, and retroperitoneal fibrosis [2], even more in rare and unusual critical situations.

As minimal invasive surgery has started to take the lead, decrease in surgical morbidity is now seen with laparoscopic and even completely intracorporeal robotic surgery [3].

The purpose of this report is to present nine typical and atypical indications for kidney autotransplantation in order to evaluate its effectiveness in maintaining kidney function and avoiding cancer recurrence and to review the current literature.

## 2. Materials and Methods

From 1999 till 2014, nine renal autotransplantations were performed in our center (Hotel Dieu de France University Hospital). A retrospective case review was done. The series included five females and four males with a mean age of 49.8 years. 4/9 patients had a solitary functioning kidney. Typical indications for autotransplantation included extended ureteric disease in 5 patients, intrasinusal tumor on a solitary kidney in 1 patient, and renal artery aneurysm in 1 patient. Atypical indications consisted in bilateral urothelial tumors in 1 patient and interrupted live kidney transplantation in 1 patient.

The kidneys were removed through a flank incision over the 11th rib or laparoscopically and transplanted to the iliac fossa. In all patients, the ureter was reimplanted in the bladder using the Lich–Gregoir technique. Mean cold ischemia time was 209 minutes.

Demographic factors, indications, renal function before and after surgery, and in the long term, cancer recurrence and disease-free survival were evaluated (Tables 1–3).

## 3. Results

**Figure 1:**
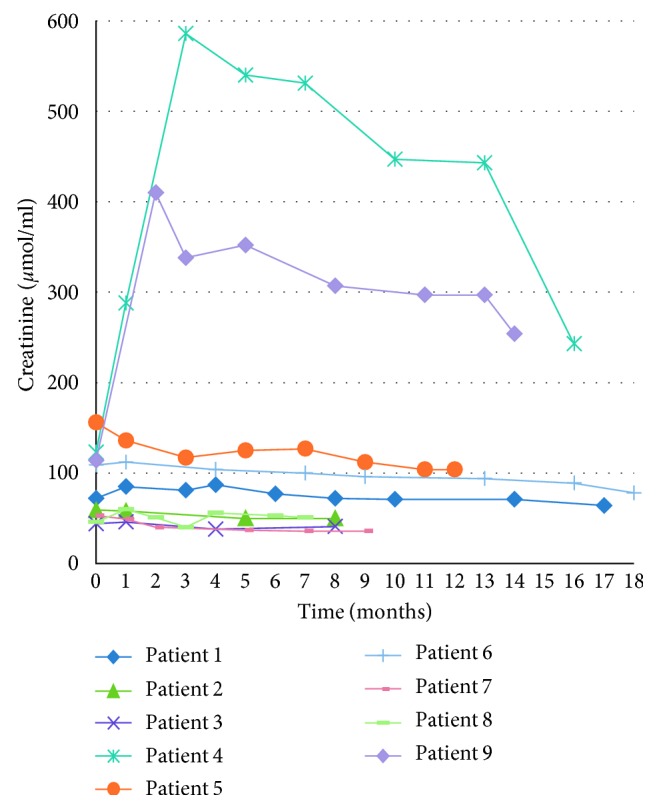
Early outcome: renal function.

Renal function was evaluated by creatinine clearance estimate using the Cockcroft–Gault equation. This was maintained in 8 patients during the early follow-up (Figure 1).

No serious complications occurred in the postoperative period. Median duration of follow-up was 50 months. In 4 patients with a normal contralateral kidney, mean preoperative and at discharge creatinine clearance were 105.45 ml/min and 121.02 ml/min, respectively. Although values showed an improvement in the kidney function, the difference was not deemed significant (*p* value 0.3).

In the other 4 patients with a solitary kidney, mean discharge creatinine clearance was 99.24 ml/min surprisingly higher than the preoperative value 96.92 ml/min. Note that the difference between these means was not significant (*p* value 0.72). At the last follow-up, kidney function was preserved for the two groups (normal contralateral kidney/solitary kidney) with relatively stable creatinine clearance values: 108.45 ml/min and 85.9 ml/min, respectively. No patients required secondary dialysis. The patient with bilateral pelvic and ureteric tumors is still cancer free, 7 years after surgery, with no recurrence on the annual CT scan. Finally, the patient with solitary kidney and intrasinusal tumor is also cancer free after 10 years of follow-up.

## 4. Discussion

The main reason for the use of kidney autotransplantation is to preserve renal parenchyma. RA is generally reserved to severe situations and is often the last option before nephrectomy. Previous studies have emphasized AR as a highly effective surgical procedure for the treatment of renal pathologies.

The most common indications of RA are the extensive ureteral lesions. In fact, it allows a direct pelvivesical anastomosis. Indeed RA is a viable option for complicated ureteral lesions and is an alternative to known methods not limited to psoas Hitch procedure, Boari flap, and transureteroureterostomy [4].

In the oncological setting, no study aimed at comparing the oncological outcome after autotransplantation versus radical surgery, but in most published papers, low recurrence rate and few complications were reported with autotransplantation in patients with renal or ureteral tumors.

The first paper was published in 1984 by Pettersson et al. [4] who performed nephroureterectomy, renal autotransplantation, and pyelocystostomy in eight patients with upper urinary tract urothelial carcinoma. Five of eight patients remained cancer free after 32 months of follow-up. Radical surgery had to be done in one patient after 4.5 years because of infiltrating tumor recurrence, and transurethral endoscopic resection was done successfully in three patients with calyceal recurrence. Pettersson et al. concluded that this procedure implies increased radicality compared with conventional conservative treatment and simplified follow-up. It may be considered in patients with bilateral tumors or tumors of a solitary kidney and in selected patients with unilateral low-grade, low-stage tumors [5].

The longest follow-up was reported by Holmang and Johansson in 2005 [6]. His study was conducted on 23 patients with urothelial neoplasm in the upper urinary tract, operated with resection and autotransplantation than followed for 7 to 20 years. Of the nine patients who had solitary kidney or bilateral tumors, three patients survived with no recurrences and no dialysis after 238 and 127 months, respectively, three patients required dialysis 2 to 9 years later, and 3 patients died of the disease. Concerning patients with normal contralateral kidney, Holmang and Johansson argued that resection and renal autotransplantation are not indicated and might even be harmful, compared to standard nephroureterectomy [6].

Ex vivo nephron-sparing surgery for renal carcinoma is also an option in solitary kidney or in bilateral tumors.

The term “bench surgery” is used to describe reconstructive surgery on diseased kidneys receiving asanguineous perfusion outside the body. The first uses of bench surgery were reported by Corman et al. in 1973. Their report and subsequent ones about bench surgery have shown how this sophisticated approach is a significant advance in urologic operative procedures.

In their report, Putnam et al. [7] reported two uses of bench surgery (staghorn calculi in a solitary kidney and abdominal aortic aneurysm with abnormalities of both renal arteries and early renal failure).

One study published in 1985 by Mayo Clinic showed similar rates of nonprogression (76%) and survival (87%) in patients with low-grade (1 or 2), low-stage (I or II), bilateral or solitary renal cell carcinoma treated with conservative surgical treatment (in situ enucleation, in situ partial nephrectomy, or an extracorporeal operation) compared to radical surgery after 5 years of follow-up [8].

Two recent studies on ex vivo nephron-sparing surgery for renal carcinoma were published in 2014.

The first series included 9 patients. The absolute indications were organ saving and technical impossibility of renal tumor resection in situ. The mean duration of operation was 297.8 minutes, and the mean period of hypothermic ischemia consisted of 112.6 minutes. A postoperative follow-up (maximum 4 years) revealed that there is not noted dissemination of the tumor, and functional condition of the transplanted kidney was satisfactory [9].

The second series included 3 patients. Two patients had complex renal cell carcinoma, and 1 patient had bilateral large angiomyolipoma. All 3 patients did not have renal replacement therapy and currently live a good quality of life. According to the authors, the advantage of this procedure over renal replacement therapy is better quality of life and cost-effectiveness [10].

In this new era of minimal invasive surgery, laparoscopic renal harvest for autotransplantation provided an opportunity to decrease morbidity. Fabrizio et al. performed the first laparoscopic nephrectomy with open autotransplantation (kidney extraction was done through a periumbilical incision followed by a transplantation through a Gibson incision) [11].

Gill et al. described retroperitoneoscopic laparoscopic nephrectomy followed by open autotransplantation using a Gibson incision for both extraction and subsequent transplantation [12].

One of the largest series of laparoscopic nephrectomies for autotransplantation was reported by Tran et al. in 2015 including 52 patients with more than 90% success rate over a 6-year follow-up period [13].

Also, although a very challenging procedure, completely intracorporeal robot-assisted nephrectomy with RA is now a feasible approach to renal preservation after major ureteral injury [3].

Finally, a randomized study comparing RA to another approach like “bowel interposition” is necessary to determine the differences in benefits and risks associated with these procedures.

Analysis of values and changes in renal clearance revealed that the majority of patients had an improvement in renal function, with the exception of two patients with a postoperative clearance of <60 ml/min (36.21 and 41.5 ml/min, resp.), knowing that these 2 patients had some degree of kidney failure before surgery (preoperative clearance of 60.5 and 59.5 ml/min, resp.). This reflects the improvement in renal function and the rarity of delayed complications.

In our study, the patient with pelvic and bilateral ureteral tumors showed remission, without any recurrence on the annual scan even 7 years after the surgery, as well as for the patient with a solitary and an intrasinusal tumor who is disease free after 10 years of follow-up.

In this study, the relatively long follow-up (average of 55 months) empowers the study and explains RAs validity and effectiveness. However, a retrospective study like ours could be criticized. The limited number of patients enrolled, the one center-one surgeon experience are some of the limitations of this study.

## 5. Conclusion

Renal autotransplantation is a rare, safe, and effective surgical procedure for the treatment of complex urologic conditions. In some instances, it may be of great utility for kidney salvage in some carefully selected patients. Our center's experience in renal autotransplantation showed low complications rate, preserved kidney function, and no cancer recurrence on the early and late follow-up.

## Figures and Tables

**Table 1:** Demographic and preoperative data in study population.

Patient	Age	Sex	BMI (kg/m^2^)	Indication for autotransplantation	Contralateral kidney
1	42	M	27.42	Extended left ureteral injury	Right kidney agenesis
2	52	F	27.34	Extended left ureteral injury	Nonfunctioning right kidney due to neglected ureteropelvic junction
3	60	F	22.8	Interrupted living kidney donor due to perioperative cardiac failure in the recipient	Normal
4	60	M	33.4	5.5 right hilum tumor (ex vivo repair)	Left nephrectomy for familial living donor
5	61	M	31.31	Right ureter fibrosis after radiotherapy for prostate cancer	Chronic renal failure
6	32	F	21.48	Left ureteral injury (stripping during ureteroscopy)	Normal
7	38	F	21.03	Right extended ureteral stenosis	Normal
8	38	F	22.58	Left renal artery aneurysm (ex vivo repair)	Normal
9	65	M	20.28	Bilateral ureteral and pelvic tumor	Nephroureterectomy for right ureteral and pelvic tumor

**Table 2:** Perioperative and postoperative parameters.

	Patient 1	Patient 2	Patient 3	Patient 4	Patient 5	Patient 6	Patient 7	Patient 8	Patient 9
Operating time (hours)	8	8	6.5	8.5	10	9	6	7.5	11
Duration of ischemia (min)	70	180	215	270	230	230	145	240	300
Duration of hospitalization (days)	17	8	7	16	12	18	9	7	14
Pre-op creatinine clearance (ml/min)	139.77	108.54	113.33	79.37	60.03	57.01	106.1	145.3	59.5
Creatinine clearance at discharge (ml/min)	157.27	109.4	123.19	40.26	90.04	76.69	153.2	131	23.6
Duration of follow-up (months)	120	6	12	60	24	72	24	120	48
Creatinine clearance at the last follow-up (ml/min)	154.29	91.52	107.24	61.6	36.21	72.68	127.78	126.1	41.93

**Table 3:** Perioperative and postoperative complications.

Patient	Complications
1	Immediate renal artery thrombosis necessitating retransplantation of the kidney in the contralateral iliac fossa
2	Urinary infection
3	None
4	Acute tubular necrosis, pneumonia
5	Intestinal occlusion medically resolved
6	Urinary infection
7	None
8	Urinary infection
9	None
